# Recent Growth of Wettability Gradient Surfaces: A Review

**DOI:** 10.34133/2022/9873075

**Published:** 2022-07-16

**Authors:** Raza Gulfam, Yongping Chen

**Affiliations:** ^1^Jiangsu Key Laboratory of Micro and Nano Heat Fluid Flow Technology and Energy Application, School of Environmental Science and Engineering, Suzhou University of Science and Technology, Suzhou 215009, China; ^2^Key Laboratory of Energy Thermal Conversion and Control of Ministry of Education, School of Energy and Environment, Southeast University, Nanjing, Jiangsu 210096, China

## Abstract

This review reports the recent progress and future prospects of wettability gradient surfaces (WGSs), particularly focusing on the governing principles, fabrication methods, classification, characterization, and applications. While transforming the inherent wettability into artificial wettability via bioinspiration, topographic micro/nanostructures are produced with changed surface energy, resulting in new droplet wetting regimes and droplet dynamic regimes. WGSs have been mainly classified in dry and wet surfaces, depending on the apparent surface states. Wettability gradient has long been documented as a surface phenomenon inducing the droplet mobility in the direction of decreasing wettability. However, it is herein critically emphasized that the wettability gradient does not always result in droplet mobility. Indeed, the sticky and slippery dynamic regimes exist in WGSs, prohibiting or allowing the droplet mobility, respectively. Lastly, the stringent bottlenecks encountered by WGSs are highlighted along with solution-oriented recommendations, and furthermore, phase change materials are strongly anticipated as a new class in WGSs. In all, WGSs intend to open up new technological insights for applications, encompassing water harvesting, droplet and bubble manipulation, controllable microfluidic systems, and condensation heat transfer, among others.

## 1. Introduction

The wettability of a pristine (originally-existing) surface is a topographic phenomenon which can be defined as the extent to which the liquids (water, oil, alcohol, acids, alkalis, etc.) interact with the topography (the upper part of the entire surface) and make it either wet or nonwet. The wettability is quantified by the contact angle [1] that a droplet makes on the topography in the equilibrium state. Based on the contact angles, a surface is deemed to own either homogeneous wettability (i.e., the contact angle is same on different positions) or heterogeneous wettability (i.e., the contact angle is not same on different positions). The heterogeneous wettability is enabled by the structural topography composed of a certain gradient, giving birth to wettability gradient surfaces (WGSs) [2, 3]. In short, WGSs can be defined as the heterogeneous surfaces having different apparent contact angles (spatial derivative) aligned asymmetrically in a certain direction, meaning that the wetting characteristics vary based on different topographical patterns (i.e., creating low wetting and high wetting zones) underneath the droplet.

The evolution of WGSs stems from the living surfaces, for example, the desert beetle [4], drain fly [5], cactus spines (consisting of both wettability gradient and physical shape gradient) [6], spider silk [7] (consisting of both wettability gradient and physical shape gradient), Salvinia molesta [8], and bamboo leaf [9]. Upon looking into the surface morphology of desert beetle, different topographical patterns are observed where the condensed water adopts the dropwise form in a primary zone (low wetting waxy zone), which then moves in the direction of a secondary zone (high wetting non-waxy zone) until it reaches the mouth [10]. In addition, the elytra of beetle consists of a near-random array of bumps on macroscopic level, as illustrated by Parker and Lawrence [10]. While, on microscopic level, the peaks of those bumps are non-waxy smooth sites, and the troughs consist of the flattened hemisphere-based waxy microstructures that are arranged in a hexagonal array, descending from elytra (top) towards mouth. This descending array, particularly when a beetle tilts itself down, plays a vital role in water droplet transport. Non-waxy sites are hydrophilic with high surface energy, while waxy sites are hydrophobic (or superhydrophobic) with low surface energy. In a word, the combined effect of wettability gradient (established by non-waxy and waxy sites of high and low surface energies) and descending array is significant in transporting the water droplets. Similarly, the bamboo leaf [9] consists of structural patterns that are different on the margin and middle. The microscopic examination depicts the large microscale papillae and nanoscale wax platelets on the margin, while comparatively small microscale papillae and nanoscale wax granules on the middle, rendering low and high wetting zones, respectively. Salvinia fern has a special kind of surface structure, consisting of a low wetting surface base onto which high wetting egg-beater-like structures exist, depicting the gradient of wettability from the tip to base. However, the dense egg-beater-like structures do not allow downward penetration of water droplets [11]. Wu et al. [12] have recently composed a comprehensive review on the bioinspired gradient surfaces and categorized the gradients generated by either wettability or geometry. The term geometry here should not be confused with morphology/geometry of topographic micro/nanostructures. Instead, this geometry specifically refers to the physical body shapes, like curvature/slope-shaped, s-shape, v-shape, t-shape, peristome, trapezia, wedge, conical, star, and spiral. Therefore, it is strongly suggested that the gradient induced by the physical body shape (geometric gradient as common in other papers) can better be renamed as physical shape gradient to avoid further confusion. It is worth-mentioning that the physical shape gradient can be obtained by cutting/carving either the substrate itself or the coating on a substrate or both. For example, Launay et al. [13] have presented the physical shape gradient by properly carving the coatings of alkylsilane and alkanethiol into wedge shapes that were indeed coated onto a silicon wafer and gold substrates. Creating the physical shape gradient by carving the substrate can be understood through the example of Ma et al. [14], in which the polyethylene substrate has been cut into trapezia shape. Most importantly, gradients can also be established by the combined effect of wettability and physical shapes, for example, the cactus spine. The physical shape of the cactus spine body is a curvature-based which has altogether low wettability (contact angle varying in the range of 20-12°) from the spine-tip (low wetting) to trichome-base (high wetting) [15], meaning that the droplet manipulation is the combined effect of slightly changing wettability gradient and largely changing conical shape. However, the physical shape gradients may not be able to provide slippery droplet dynamics unless it is coated with other slippery materials [14]. Also, Xiao et al. [16] have explicitly reported that the geometric gradient surfaces suffer from drawback of long-distance transportation of gas bubbles in the aqueous environment. To sum up, WGSs exist in nature that have inspired the researchers, assisting construct the similar topographic patterns on the metallic sheets [17], metallic wires [18], and polymer-based [19] substrates, leading to artificial WGSs as exposed hereinafter.

In this paper, WGSs are focused, addressing their present evolution and scope in the future. The brief background of the fundamental surface science has been provided, interpreting the droplet wetting and dynamic regimes, as well as their evolution from the living surfaces to the non-living surfaces (Section 2). In particular, the governing principles of wettability gradient and droplet dynamics of WGSs are elaborated (Section 3). WGSs are carefully classified in dry and wet classes, followed by the characterizations and applications (Section 4). The loopholes regarding the complexity of fabrication of WGSs have been uncovered, and the recommendations are presented based on alternatively convenient methods, personal perspectives, and engineering insights. Also, phase change materials are discussed together with emphasizing their active usage in the fabrication of WGSs in the future (Section 5).

## 2. Bioinspired Transformation and Topographic Structures

The surfaces can be broadly categorized into living (e.g., lotus leaf, rose petal, fish, butterfly wings, and beetle) and nonliving surfaces (e.g., metals, woods, fabrics, and glasses). Particularly in the last several decades, mankind started pondering over the interaction of water with the living surfaces, stimulating many questions across the globe, for example, why the rain water cannot wet the wings of a butterfly, why the water droplet sticks to the vertical rose petals, why the water droplet speedily moves on slightly tilted lotus leaves, how the condensed water moves from the back of desert beetle towards mouth, and how the insects are slipped down and entrapped in the pitcher plant. These questions had been gradually answered both scientifically and logically in the light of wetting regimes. Surface topographies of most bioorganisms were carefully examined under advanced microscopes, discovering the surface scaffold composed of micro/nanostructures of various geometries, as well as epicuticular waxy layers surrounding the micro/nanostructures [20]. Additionally, there are many factors controlling the interaction of water and droplet penetration into the micro/nanostructures of living surfaces. Fundamentally, micro/nanostructures of various geometries, their certain arrangements, surface chemistry, and epicuticular waxy layers surrounding those micro/nanostructures are supposed to be the most important. The epidermal waxy layer itself further consists of micro/nanostructures varying in numbers, sizes, shapes, and stomatal densities, leading to different simple structures or unique hierarchical structures building the isotropic, anisotropic, symmetrical, or asymmetrical patterns [21].

**Figure 1:**
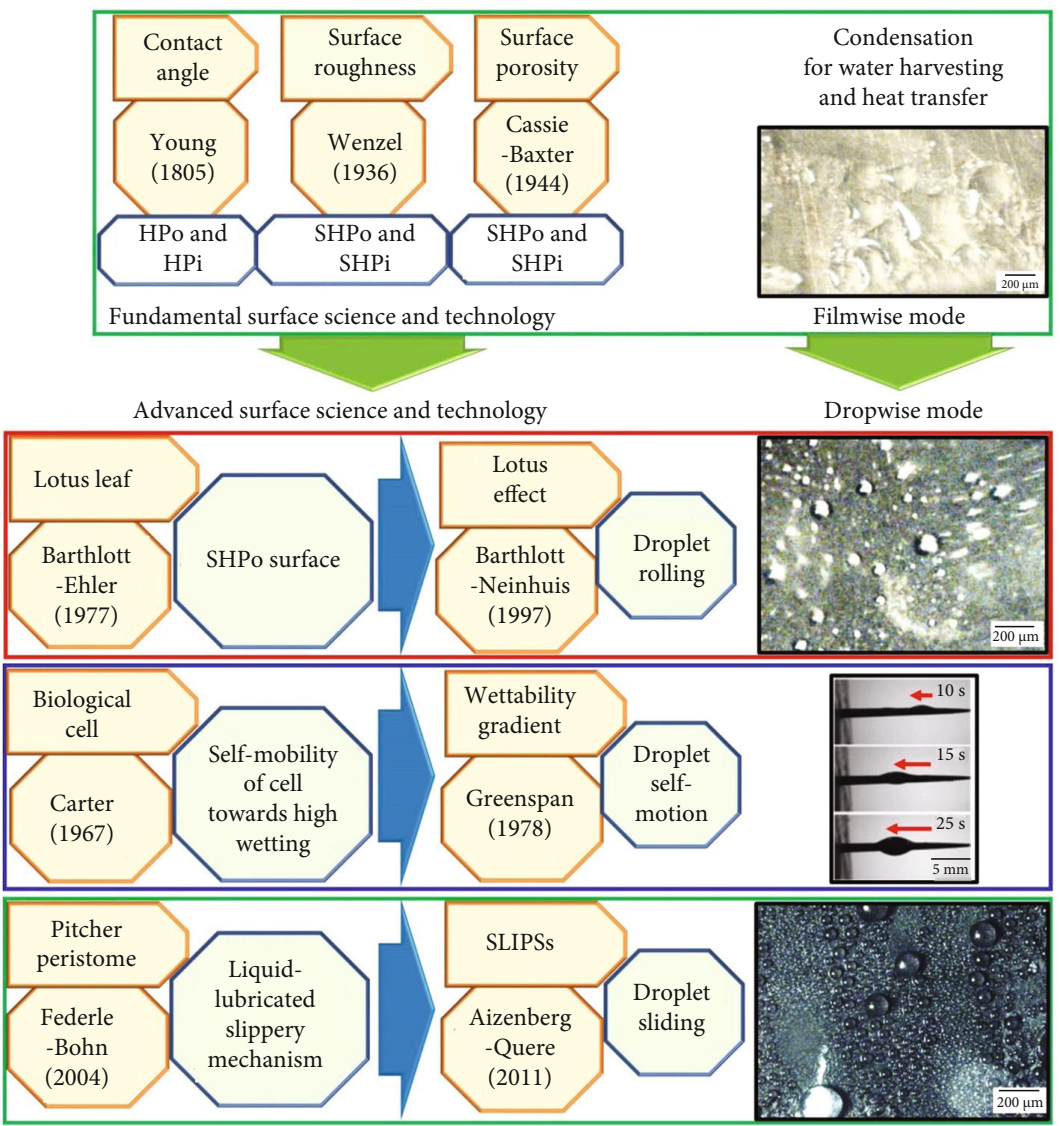
Evolutionary timeline of the fundamental surface science and technology to the advanced surface science and technology (note: HPo, HPi, SHPo, and SHPi stand for hydrophobic, hydrophilic, superhydrophobic, and superhydrophilic, respectively. Images on the condensation of water vapors belong to the authors' reference [24], except for the conical wire that belongs to Reference [18]).

Shown in Figure 1 is the historical evolutionary timeline of the surface science and technology. A gradual but constant progress can be seen, starting from the model developments (Young's model, Wenzel's model, and Cassie-Baxter's model), parameter introductions, factor extensions, mechanism establishments, and deep observations on the living surfaces, resulting in the advanced surface science and technology. Referring to Figure 1, with Young's contribution back in 1805, the flat [22] or smooth [23] surfaces have been quantified via Young's contact angle *θ*_e_ (°), presenting the fundamental relationship of the wetting via interfacial surface energies *γ* of solid-vapor (sv), solid-liquid (sl), and liquid-vapor (lv) as presented in Equation (1) [23]. Later in 1936, Wenzel contributed to define the roughened surfaces by providing the concept of roughness *R* that resulted in the apparent contact angle *θ*_a_ (°) on the projected area (Equation (2) [23]), while in 1944, Cassie-Baxter put forwarded the concept of surface porosity in the roughened surfaces via solid fraction *f* (Equation (3) [23]). In the light of Wenzel and Cassie-Baxter's theories, the effect of micro/nanostructures and surface energy both play a significant role in controlling the wetting.

(1) $$\begin{array}{c} \cos\theta_{\text{e}}=\frac{\gamma_{\text{sv}}-\gamma_{\text{sl}}}{\gamma_{\text{lv}}}, \end{array}$$

(2) $$\begin{array}{c} R=\frac{\cos\theta_{\text{a}}}{\cos\theta_{\text{e}}}, \end{array}$$

(3) $$\begin{array}{c} \cos\theta_{\text{a}}=f_{1}\cos\theta_{\text{e}}-\left(1-f_{1}\right). \end{array}$$

**Figure 2:**
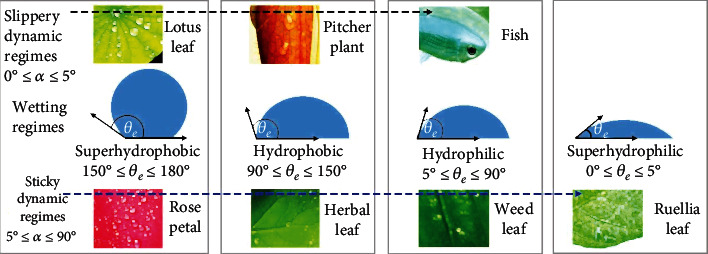
Classification of droplet wetting and droplet dynamic (sticky and slippery) regimes. The examples of living surfaces demonstrate the slippery dynamics (top) and sticky dynamics (bottom) (note: *θ*_e_ (°) is the equilibrium contact angle and *α* (°) is the sliding/rolling angle).

On the one hand, the living surfaces bear four droplet wetting regimes depending on the extent of droplet penetration into the micro/nanostructures, namely, the hydrophilic wetting regime (moderate wetting), superhydrophilic wetting regime (complete wetting), hydrophobic wetting regime (the low wetting), and superhydrophobic wetting regime (lowest wetting), which are quantified by the equilibrium contact angle *θ*_e_ (°), as summarized in Figure 2. Based on the interfacial adhesion between water and living surfaces, two droplet dynamic regimes (each corresponding to the droplet wetting regimes) can be categorized, such as the sticky and slippery (Figure 2), prohibiting or allowing the droplet mobility, respectively. The droplet dynamic regimes can be quantified by measuring the sliding/rolling angles *α* (°), referring to the angle of inclination of the surface at which droplet starts moving [25]. The droplet dynamics is strongly influenced by the waxy layer and its micro/nanostructures which are thus responsible for the sticky and slippery dynamics. For instance, the biological geometry of lotus leaf and pitcher plant consists of nanoscaled hairy and plate-like structures, respectively, that are additionally covered by a thin waxy layer [26], imparting the slipperiness against water droplets.

On the other hand, the nonliving surfaces distinctively bear two droplet wetting regimes, i.e., hydrophilic (e.g., silicon surface [27]) and hydrophobic (e.g., paraffin wax surface [28]), while they inherently bear one droplet dynamic regime which is mostly sticky.

With the advent of technology, the understanding has progressively emerged to copy the surface scaffold of the living organisms and create them upon the nonliving surfaces. This process of mimicking the surface scaffolds is known as bioinspiration, resulting in the smart surfaces with artificial wettability having different droplet wetting regimes and unique droplet dynamic regimes. For example, the surface scaffold of the lotus leaf has been copied to artificially fabricate the bioinspired surfaces known as superhydrophobic surfaces [29] where the solid-liquid interface is based on the dry interaction providing the rolling mechanism during droplet mobility, while the surface scaffold of the pitcher plant has been copied to realize the bioinspired surfaces, widely known as slippery liquid-infused porous surfaces (SLIPSs), where a wet solid-liquid interface is established providing the sliding mechanism during droplet mobility [24].

In the initial step of bioinspired mimicking, the inherent wettability of pristine surfaces is converted into artificial wettability by various surface engineering methods, resulting in either extruding or intruding topographic micro/nanostructures. Extruding topographic micro/nanostructures are developed onto the pristine surface commonly through physical/chemical coating, existing as the protrusions arranged either randomly or regularly, e.g., coating of Cu_2_O/CuO [30] or film of copper sulfide [31] onto the copper substrate.Whereas, intruding topographic micro/nanostructures are inscribed into the pristine topography through scratching, ploughing, or tunneling, existing as the engraved structures also arranged either randomly or regularly, e.g., microholes in polydimethylsiloxane (PDMS) surface [32].

In the final step of bioinspired mimicking, the structural topography is refined by the artificial coatings, fabricated through thermal processes or physical/chemical coatings, which impart the artificial characteristics of either hydrophilicity, superhydrophilicity, hydrophobicity, superhydrophobicity, ominiphobicity, or superomniphobicity. The artificial coatings can be obtained by employing a wide variety of coating materials, which are normally categorized depending on the surface energy (or surface tension), including the low surface energy materials providing hydrophobic/superhydrophobic wetting regime (e.g., perfluoroalkylsilane, perfluorooctyltriethoxysilane, Teflon, and alkyltrichlorosilane, [33–35]), as well as high surface energy materials providing hydrophilic/superhydrophilic wetting regime (e.g., titanium dioxide and silicon dioxide [36]), etc. Although the artificial coatings are promisingly helpful in altering the inherent wettability and surface energy ensuring the novel regimes, the instability and short lifespan [37, 38] of the coating materials is the hitch of major concern.

## 3. Governing Principles of WGSs

### 3.1. Governing Principle of Wettability Gradient

Historically, the identification of wettability gradient is dated back to 1967 when Carter first discovered the self-mobility of a biological cell, followed by further attempts on its mechanism by Greenspan in 1978 (modeling and experiments) [39], as well as by Brochard in 1989 (modeling) which were further experimentally demonstrated with explicit evidences by Chaudhury and Whitesides in 1992 [40, 41]. It is of broad importance to put special emphasis on the gradient in surface science, which is herein defined as the spatial variable of a surface parameter with a magnitude and certain direction. The apparent contact angle is the most widely used surface parameter of which the spatial derivative, spanning from the superhydrophobic to superhydrophilic, superhydrophobic to hydrophobic, and hydrophobic to hydrophilic regimes, establishes the wettability gradient *W*_g_ for WGSs as follows [42]:

(4) $$\begin{array}{c} W_{\text{g}}=\frac{\text{dcos}\theta_{\text{a}}}{dx}. \end{array}$$

Wettability gradient can be achieved by arranging the wetting regimes in several ways, for example, periodic arrangement, asymmetric stepwise arrangement, patterned arrangement, and arrangement along certain shapes such as triangular, radial, or diagonal. Every arrangement refers to the directional change of the apparent contact angles (spatial variable) lying within a certain framework positioned horizontally or vertically. For example, in periodic arrangement, small patches providing the gradient from hydrophobic to hydrophilic regimes are repeated several times; in patterned arrangement, hydrophilic stripes and hydrophobic stripes are repeated one after other; in asymmetric stepwise arrangement, the steps providing the gradient are positioned in a certain decreasing or increasing alignment.

It should be noted that different apparent contact angles can also be obtained by changing the surface roughness or using different composition-based chemical coatings and motivated by these facts; few authors also utilize the roughness gradient on the patterned arrangement [43] or topographic gradient [44] or chemical gradient [45] to interpret the wettability gradient. The chemical gradients can be realized by using the combination of chemicals having different wetting regimes based on the gradually varying compositions along the length. Meanwhile, the roughness is an important surface parameter that depends on the inherent wetting regime of the employed surface (Wenzel theory). For example, when the roughness of hydrophilic surface increases, the surface becomes more hydrophilic [46], while when the roughness of hydrophobic surface increases, the surface becomes more hydrophobic [46]. However, the role of topographic micro/nanostructures is more influential to control the wetting regimes compared with the surface roughness, and the single criterion of the apparent contact angle in a certain direction (spatial derivative) can be used to quantify the wettability gradient.

### 3.2. Governing Principle of Droplet Dynamics

**Figure 3:**
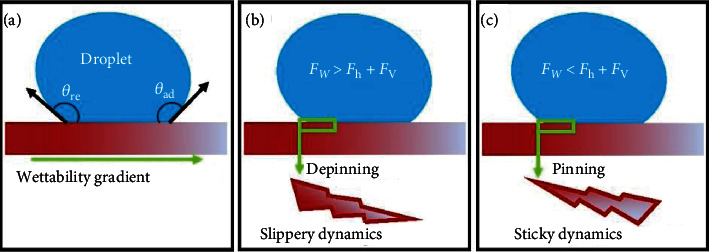
Mechanism of droplet dynamics influenced by the involved forces: (a) droplet affected by the wettability gradient, (b) slippery dynamics, and (c) sticky dynamics.

WGSs consist of several wetting regimes on the same surface. The droplet dynamics and wettability gradient may be interlinked (Figures 3(a)–3(c)), such as to say that the droplet mobility should occur in the direction of decreasing wettability (i.e., superhydrophobic to superhydrophilic wetting regimes) [41, 47]. However, the decreasing wettability in a certain direction cannot always ensure the droplet mobility, because the wetting regimes can be either sticky or slippery depending on the contribution of topographic micro/nanostructures and surface energy. Briefly, for the efficient droplet dynamics, the wettability gradient should necessarily be established in the slippery wetting regimes. During the droplet mobility particularly on WGSs, the droplet encounters three main forces [42], i.e., wettability force (*F*_w_) that is indispensable to drive the droplet influenced by the wetting regimes and the underlying surface energy at the solid-liquid interface; hysteresis force (*F*_h_) that is caused by contact angle hysteresis which tends to oppose the droplet movement; and viscous force (*F*_v_) that is caused by the viscous stress at the solid-liquid interface inducing internal resistance within the liquid during droplet movement. The equations of forces are listed as follows [42]:

(5) $$\begin{array}{c} F_{\text{w}}=\gamma_{lv}\pi r^{2}\frac{d\cos\theta_{\text{a}}}{dx}, \end{array}$$

(6) $$\begin{array}{c} F_{\text{h}}=2\gamma_{\text{lv}}r\left(\cos\theta_{\text{ad}}-\cos\theta_{\text{re}}\right), \end{array}$$

(7) $$\begin{array}{c} F_{\text{v}}=\int_{L}^{R}\sigma_{xz}\left(0\right)fRdx, \end{array}$$

where *γ*_lv_ is the surface tension of droplet at the liquid-vapor interface, *r* is the base radius of droplet, *θ*_a_ (°) is the apparent contact angle, *θ*_ad_ (°) is the advancing contact angle, *θ*_re_ (°) is the receding contact angle, *σ*_*xz*_(0) is the viscous stress at the solid-liquid interface, *f* is the solid fraction, and *R* is the roughness of flat region. The lower limit *L* and upper limit *R* denote the left and right ridges of the droplet (for further details, see the supporting information of Reference [42]), respectively. Equations (5) and (6) reveal the critical contribution of wettability gradient and contact angle hysteresis on the droplet dynamics in WGSs, respectively.

When the wettability force (*F*_w_) becomes dominant over the combined effect of *F*_h_ and *F*_v_, i.e., *F*_w_ > *F*_h_ + *F*_v_, the droplet undergoes the depinning phenomenon and moves on WGSs providing slippery dynamics (Figure 3(b)), while the reverse holds true for the sticky dynamics, i.e., *F*_w_ < *F*_h_ + *F*_v_ (Figure 3(c)). In particular, the asymmetric impact across the advancing and receding ridges [48] is deemed to be necessary for the droplet mobility, e.g., creating the wettability gradient from the less hydrophilic regime (low surface energy) to the more hydrophilic regime (high surface energy) [49].

In summary, the governing mechanism of droplet dynamics on WGSs is the combination of various factors, encompassing but not limited to the wetting regimes and the corresponding surface energies [49], the surface tension of droplet [48], force imbalance and asymmetric impact at the contact line [50], droplet volume [51], gradient-enabled self-response [52], and/or the gradient-stimulus-combined response (external driving force). However, the external driving force on WGSs can also be fully exempted as recently reported in some cases [43, 53], i.e., if the structural growth is sufficiently tuned such that the region-region width is optimized, the droplet dynamics can be rendered promisingly self-stimulus (i.e., self-propelled) even on the horizontally aligned WGSs. The feature of self-stimulus droplet dynamics renders WGSs as innovative candidates among slippery surfaces. However, the commonality between WGSs and other slippery surfaces is the usage of artificial coating, which is declared to be an indispensable layer if the efficient droplet dynamics is the main scientific objective. Therefore, with wettability gradient as the primary driver [53] and artificial coating as the supporting driver, droplet from region of the highest apparent contact angles (the lowest wettability depicting phobic regime) has the capability to move based on the low surface energy solid-liquid interface. When that droplet approaches the region of relatively lowest apparent contact angles (the highest wettability depicting philic regime), the anterior dynamic energy is utilized to overcome the high surface energy solid-liquid interface, providing the net energy sufficient for the self-mobility onward. On a slippery surface, the surface tension [47] of droplet itself encounters imbalance while transiting from one wetting regime (say superhydrophobic) to second adjacent wetting regime (say hydrophobic) adopting the asymmetric shape, thereafter paving the ways for droplet motion. Indeed, the surface energy plays an influential role in establishing a wettability gradient, though it is hard to specify the role of surface energy for WGSs in general. But in particular, it can be specified separately for the sticky and slippery dynamic regimes. The sticky regime is prone to induce high surface energy disallowing the droplet mobility, while in the slippery regime, surface energy is supposed to vary from the highest to the lowest, i.e., superhydrophobic/hydrophobic to hydrophilic/superhydrophilic, depending on the topographic micro/nanostructures in the wetting regimes.

Meanwhile, the droplet also transits through several mobility mechanisms, including the rolling mechanism when the liquid droplet interacts with a dry slippery surface (superhydrophobic/hydrophobic surface), the droplet spreading mechanism when the liquid droplet interacts with a dry sticky surface (hydrophilic regime), and the sliding mechanism or combined spreading-sliding mechanism when the liquid droplet interacts with a wet slippery surface (hydrophobic or hydrophilic SLIPSs).

## 4. Fabrication, Classification, Characterization, and Applications of WGSs

Until recently, WGSs have been fabricated through many methods, necessitating different constraints and providing different configurations, as listed in Tables 1 and 2. The most common methods, namely, gradual solution-rise/gradual solution-removal/gradual substrate move methods as well as vapor diffusion/physical vapor deposition methods, are defined and exemplified underneath.

In gradual solution-rise method, the substrate is hanged in the beaker followed by slow addition of solution such that it can immerse the surface region by region under variable contact time. After several moments of contact time against every region, the more solution is dropped into the beaker, causing to raise the solution level that can immerse the substrate region lying above the previously dipped bottom region. Continuing this way, the desired length of the substrate is made to interact with the solution step by step depending on solution-addition speed, leading to develop a gradient from the bottom to top [46]. The other similar way is the gradual solution removal method wherein the beaker is filled with a solution and the substrate is immersed in it. From the bottom, the solution is removed through a valve interval after interval, providing the different contact time, i.e., the solution at the top deems to spend a short period of contact time, and along downward, the contact time deems to be increased. Consequently, the gradient can be developed from the bottom (high gradient) to the top (low gradient) [54].

In gradual substrate-move method, the beaker with a constant level of solution is filled, and then, the substrate is either moved down or pulled up step by step under variable contact time necessary for each respective region, also creating a gradient from the bottom to top [55]. The underlying mechanism is the contact time-dependent relative interaction of the solution with different regions of the substrate, i.e., the bottom region interacts with the solution for the longest time, the upper adjacent region relatively interacts for a longer time, while the upper-most adjacent region relatively interacts for the least time, and so on until the whole substrate is immersed. Corresponding to the contact time, the solution moieties can be coated differently at various regions due to which the relative wettability gradient is achieved thereupon. Zhao et al. [56], prepared WGS on copper substrate by slowly moving it into the precursor solution, obtaining the gradient along the dipping direction. Gradual solution-rise or gradual substrate-move methods can be conducted either to develop a physical coating [46] or chemical coating [54] on the substrates.

In vapor diffusion, the substrate is exposed to the solution lying underneath. The solution is made to evaporate, while the substrate is slowly moved allowing different interaction time at the adjacent regions. Thereby, vapors are diffused onto the substrate, leading to the wettability gradient of different types, such as linear and radial gradients. Vapor diffusion can also be performed by locally evaporating the solutions applied on the substrates, providing the patterned arrangement [47]. For example, Zhao et al. [56], attempted preparing the successful radial wettability gradient by putting a solution drop on the copper substrate and letting it evaporate under fume hood.

Physical vapor deposition can be performed through several ways, such as with help of thermal process, laser light, and plasma-enhanced process. Thermal process is almost similar as described above in case of vapor diffusion method. In laser light process, the desired material is locally vaporized through high-energy laser beam that is simultaneously deposited onto the substrate. Abbasiasl et al. [57] prepared WGS on the copper substrate by depositing the hydrophobic chromium on superhydrophobic background with help of electron beam physical vapor deposition.

**Figure 4:**
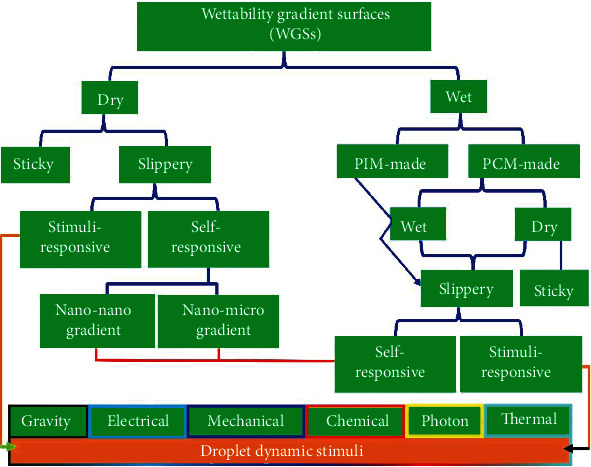
Classification of WGSs.

Guided by the influential topographic states, WGSs are mainly classified into two types, namely, dry WGSs and wet WGSs, as demonstrated in Figure 4.

Dry WGSs establish the contact with the liquid droplet through a dry interface, which can be further divided into sticky and slippery classes. An example of dry WGS [58] is the formation (of concentric nanopillars) onto the cyclic olefin copolymer substrate, consisting of a central hydrophobic wetting regime which tends to be less hydrophobic towards the edges.

Wet WGSs have been recently presented by taking inspiration from the slippery liquid-infused porous surfaces (SLIPSs). Their topographic micro/nanostructures are additionally impregnated with different slippery materials through which the wet interface is established. The slippery materials are basically of two types. One type is the phase invariant materials (PIMs), including slippery oils such as silicone oils and Krytox oils. Other type is the phase change materials (PCMs) (listed in Table 3) that can provide different levels of adhesions and slipperiness, depending on their physical phases. By controlling the size scale of topographic micro/nanostructures or infusing the slippery materials into micro/nanostructures of WSG, they can be rendered responsive to various dynamic stimuli, encompassing self-responsive, electroresponsive, gravity-responsive, photoresponsive, chemoresponsive, etc., as illustrated in Figure 4.

### 4.1. Dry WGSs

#### 4.1.1. Sticky Dry WGSs

**Figure 5:**
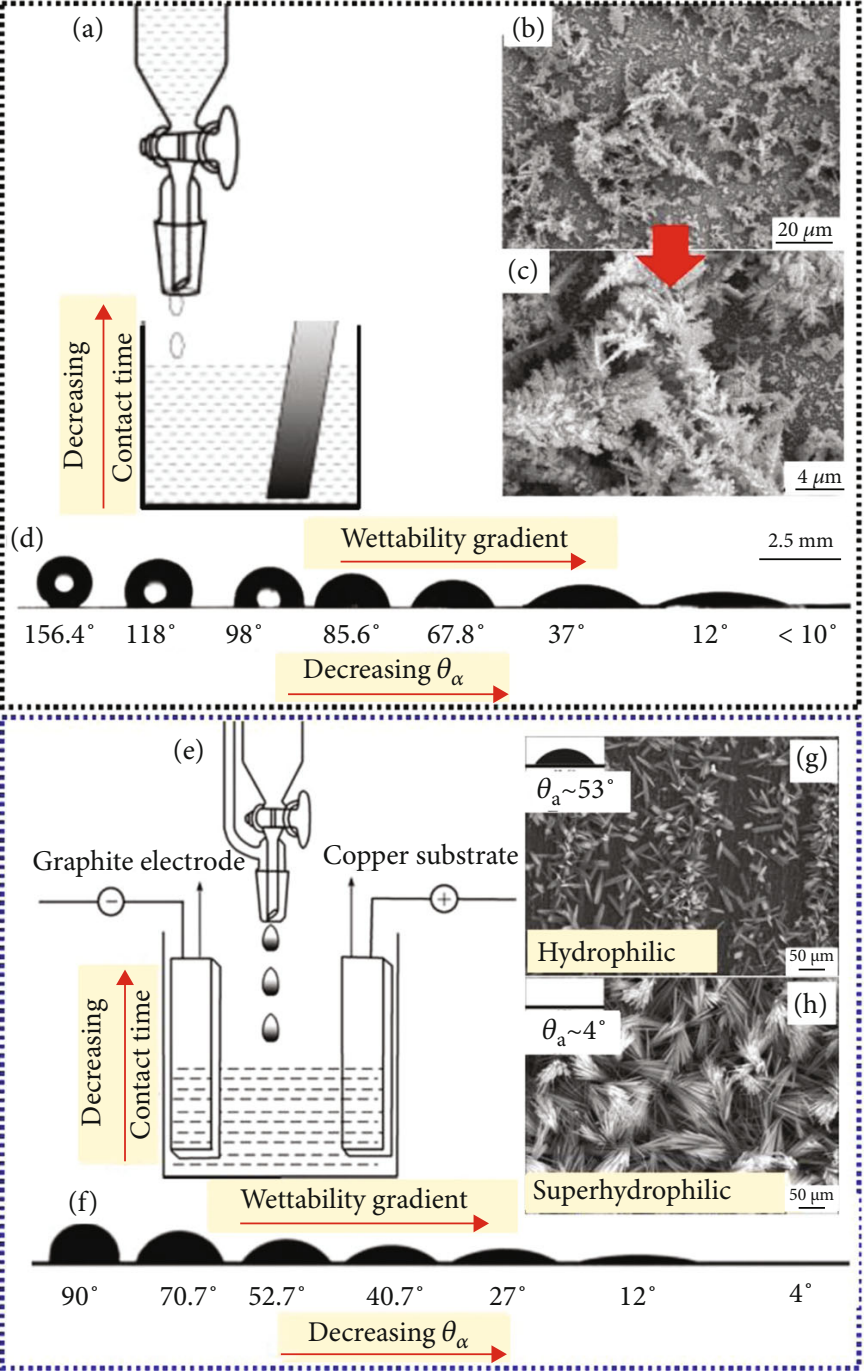
(a) Gradual solution rise method, (b, c) surface morphology, (d) wettability gradient [46], (e) gradual solution rise combined with electrochemical oxidation method, (f) wettability gradient, and (g, h) surface morphology and wetting regimes [59].

The gradual solution-rise method has been adopted to realize the wettability gradient between two extreme wetting regimes, i.e., superhydrophobicity to superhydrophilicity [46]. The experimental setup is depicted in Figure 5(a). First of all, the pristine gold surface has been roughened through the electrodeposition of gold. Subsequently, the dilute solution of HS(CH_2_)10CH_2_OH has been slowly added with a controlled speed to the test beaker where the rough gold substrate (Figures 5(b) and 5(c)) was vertically hanged, leading to the adsorption of thiol molecules. With the optimum immersion time of 10 min and solution concentration of 1 mmol/L, the rough gold substrate exhibited a superhydrophobic wetting regime (*θ*_a_ > 150°) and slippery droplet dynamic regime (rolling angle of less than 5°). To realize the wettability transition thereof, the solution concentration has varied between 1 mmol/L and 0.05 mmol/L, resulting in variable adsorption dynamics of thiol molecules that facilitated the wettability gradient encompassing the position-oriented range of 156° ≤ *θ*_a_ ≤ 10°, as illustrated in Figure 5(d). However, the wettability gradient does not support the droplet mobility along the whole substrate, drastically impacting the droplet dynamics and reserving it between two extreme regimes, either sticky or slippery, i.e., droplet mobility can only be observed in the superhydrophobic regime.

By combining the gradual solution-rise method with electrochemical anodization process [59], the wettability gradient has also been established on copper foil spanning between hydrophilic-superhydrophilic wetting regimes. An electrolytic cell has been designed consisting of a negative graphite-based electrode and positive copper-based electrode at which the coating is targeted, as depicted in Figure 5(e). The aqueous solution of KOH has been employed as an electrolyte, which was added to the electrolytic cell slowly with certain addition speed such that the different regions of the copper foil could develop different contact times, resulting in different growth rates of Cu(OH)_2_ nanoribbons and establishing the wettability gradient from the top to the bottom (Figure 5(f)). As a consequence, with a small contact time between solution and surface at the middle, the wettability tends to be hydrophilic *θ*_a_ ~ 53° with the corresponding surface morphology in Figure 5(g). The wettability of the coated copper foil becomes superhydrophilic (*θ*_a_ ~ 4° with the corresponding surface morphology in Figure 5(h)) at the bottom that is ascribed to the longest contact time. Although the wettability gradient in droplet wetting regimes has been effectively obtained, however, the droplet dynamic regimes have not been reported [59]. It is thus concluded that the wettability gradient can be favorable for efficient droplet dynamics particularly if the underlying topographic micro/nanostructures are well-homogenized with an intact artificial coating; however, these aspects have not been elucidated by Yu et al. [46] and Cheng et al. [59].

#### 4.1.2. Slippery Self-Responsive Dry WGSs

To realize the slippery self-responsive droplet dynamics, WGS based on the patterned arrangements has been fabricated on a silicon wafer via laser etching method [60]. The achieved wettability gradient swipes between superhydrophobic and hydrophobic wetting regimes that is mainly governed by the variation of position-oriented surface roughness (also named as roughness gradient). The surface roughness has been maintained by carving the grooves of different geometries, encompassing vertically aligned, concentric circular, horizontally aligned, and pillar-shaped. In each geometry, the roughness gradient has been attained by controlling the groove spacing and groove density at various regions. However, droplet dynamics has still encountered the mobility challenges due to very high roughness ensuing the absolute wetting of the surface, which have been resolved by developing the artificial coating via fluoroalkylsilane. Consequently, the continuous droplet mobility has been observed, concluding that the efficient droplet dynamics is therefore the result of the combined interplay of the wettability gradient and artificial coating.

**Figure 6:**
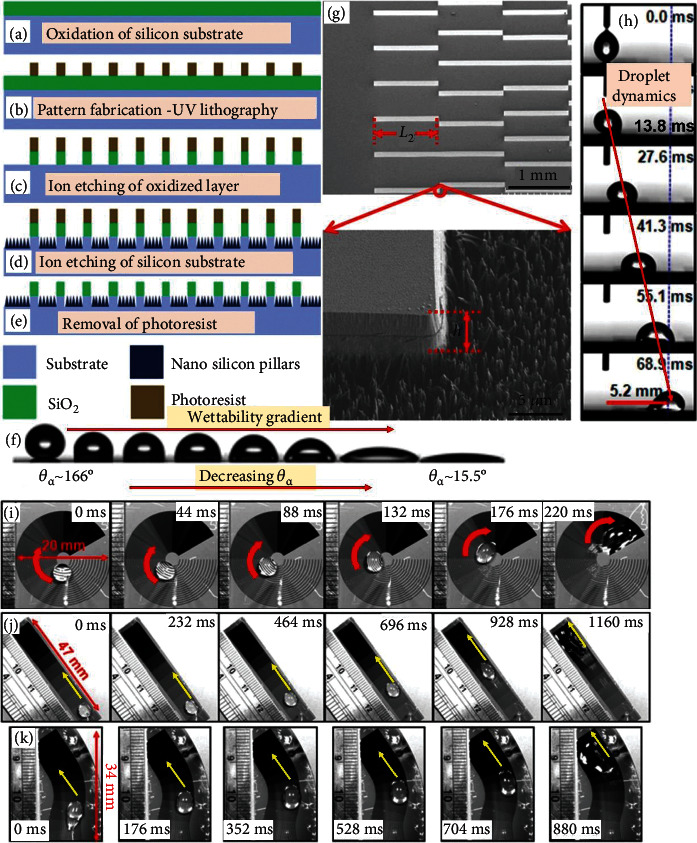
(a–e) Preparation steps of dry WGS, (f) wettability gradient, (g) surface morphology, (h) droplet dynamics, and self-stimulus uphill droplet transport along (i) circular track, (j) straight track, and (k) S-shaped track [42].

Achieving the self-stimulus droplet dynamics is of great interest, particularly for remote applications. WGSs are the promising candidates allowing the droplets driven by the structural topography and varying surface energy, meaning that external energy either via an active response or gravitational force (substrate tilting) is not required, which is called self-stimulus. For example, WGS (patterned arrangement) has been fabricated on a silicon glass wafer [42], consisting of silicon nanopillars residing in the valleys enclosed by the silicon microstripes. Nanopillars have been fabricated via the deep reactive ion etching method. The detailed fabrication method has been carried out in sequential steps as depicted in Figures 6(a)–6(e). Nanopillars ensure the superhydrophobic wetting regime (*θ*_a_ of 166°), while the microstripes offer the hydrophilic wetting regime (*θ*_a_ of 15.5°), i.e., wettability gradient has been established from nanopillars to microstrips, as can be seen in Figures 6(f) and 6(g). The force analysis depicted that the nanostructures (nanopillars) played the critical role in reducing the resistance force and, in return, providing the large driving force that enables the self-responsive dynamics. Meanwhile, the length of microstripes has also been found crucial in controlling the droplet dynamics. The droplet dynamics are depicted in Figure 6(h). In addition, WG-oriented three paths, namely, annular (Figure 6(i)), straight-uphill (Figure 6(j)), and S-shaped (Figure 6(k)), have been developed over which the self-mobilized droplet dynamics are successfully revealed, i.e., no gravitational energy or active stimulus was provided. This reasonably shows the great potential of WGSs that can help bring the technological revolution in the surface science both on an academic and industrial scale.

#### 4.1.3. Slippery Stimuli-Responsive Dry WGSs

**Figure 7:**
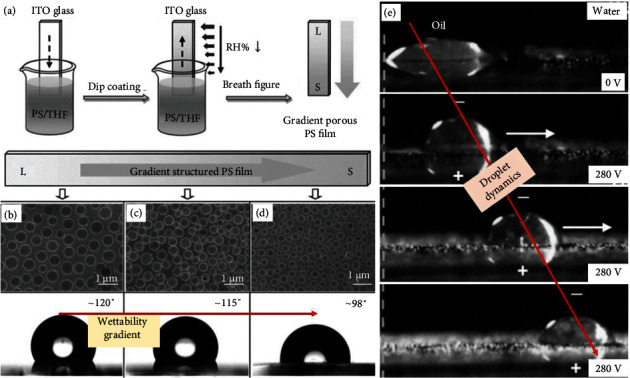
(a) Preparation of WGS, (b–d) position-oriented surface morphology and wettability gradient, and (e) droplet dynamics [55].

WGS [55] has been built on the ITO-based glass substrate by coating the polystyrene via the graduate substrate-move method (Figure 7(a)). The microscopic examination notably depicts the size-dependent pores becoming saturated from the top (L side) to the bottom (S side containing more small number of pores), which is therefore accountable for maintaining the microstructural gradient that is further supported and confirmed by the reasonable change of apparent contact angle from ~120° to ~98° (more hydrophobic to less hydrophobic wettability gradient) (Figures 7(b)–7(d)).

The droplet dynamics have been demonstrated under the influence of inclined surface and electric potential (Figure 7(e)). Based on the gradient of polymeric microstructures arranged from the L to S region, the mobility of water droplet occurred (sliding angle of 9°) from more hydrophobic region (L) towards less hydrophobic region (S) under the mechanism of asymmetric droplet shape. In addition, the electric potential-responsive underwater oil droplet movement has been exhibited by merging WGS between positive and negative electrodes, as shown in Figure 7(e).

#### 4.1.4. PCM-Coated Dry WGSs

**Figure 8:**
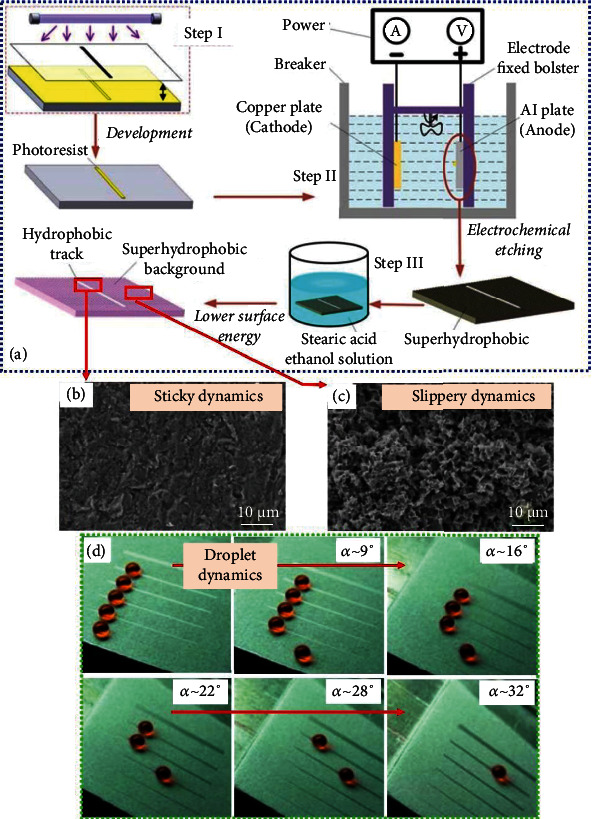
(a) Peparation of WGS, (b, c) surface morphology, and (d) droplet dynamics [61].

WGS with low and high adhesions have been presented [61] by modifying the aluminum sheets sequentially via lithography (step 1 in Figure 8(a)), electrochemical etching (step 2 in Figure 8(a)), and developing the PCM-based artificial coating (PCM: stearic acid) (step 3 in Figure 8(a)), leading to sticky and slippery droplet dynamic regimes.

The wettability gradient has been established by constructing the width-dependent sticky hydrophobic tracks on the slippery superhydrophobic background [61]. The effect of surface roughness and track width on the droplet dynamics has been elucidated such that, even with artificial coating, microroughness (see Figure 8(b)) of the tracks strictly brings about a sticky regime, while the micro/nanoroughness (see Figure 8(c)) of the background surface creates the slippery regime activating the droplet mobility depending on the droplet volume as well as on the sliding angles. The droplet dynamic regimes are depicted in Figure 8(d). The underlying slippery mechanism is enabled at the penalty of the largest droplet volume and the lowest width track, i.e., when the width track is the lowest possible (very small sticky hydrophobic regime), the largest water droplet intends to link more with the slippery superhydrophobic regime, thus droplet mobility is realized.

Besides the above-mentioned dry WGSs, the additional summary is listed in Table 1.

### 4.2. Wet WGSs

#### 4.2.1. Slippery Stimuli-Responsive PIM-Infused Wet WGSs

**Figure 9:**
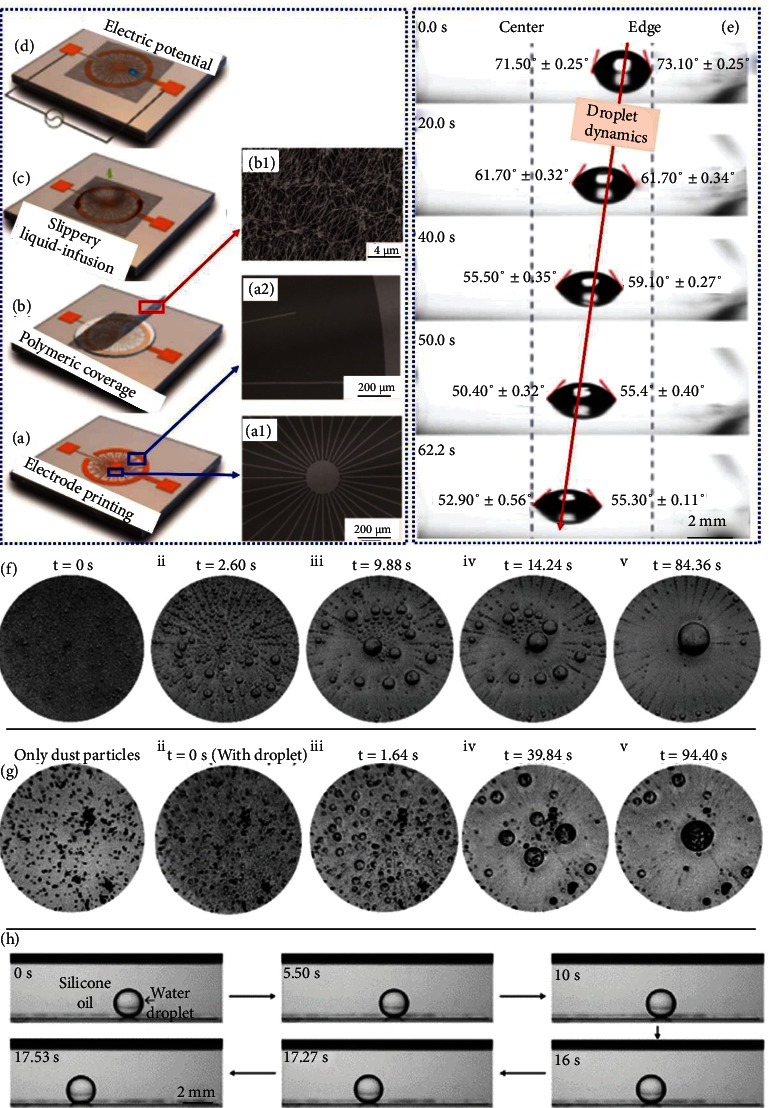
(a–d) Fabrication of WGS, (e) droplet dynamics, and applications of WGS in (f) collection of droplets, (g) self-cleaning, and (h) under-oil water droplet transport [84].

Wet WGSs indeed resemble SLIPSs as both make use of the slippery liquids. A novel approach has been envisioned on stimuli-responsive slippery liquid-infused (SLI) WGS, anticipating the considerable potential of the droplet dynamics induced by electric potential (i.e., substrate tilting is exempted). On the glass substrate [84], ITO-based (indium tin oxide) electrodes at various angles have been radially fabricated via the printing-infusion method, as depicted in Figures 9(a)–9(d). Further, the electrodes are surrounded with polymeric film (polytetrafluoroethylene) with two functions, i.e., insulating the electrodes, as well as creating the porous scaffold into which a slippery liquid (Krytox GPL oil-103) has been impregnated, resulting in electrowetted SLI-WGS. When an electric potential is applied to that SLI-WGS, the radially positioned angles cause to produce the wettability gradient from the edges towards the center necessary for the dynamics of oil droplets. Illustrated in Figure 9(e) is the demonstration of droplet movement under the influence of 900 V and 60 kHz. The achieved wettability gradient on SLI-WGS can better be understood with the help of electrowetting and dielectrowetting theories, stating that the change in contact angles of droplet is proportional to the applied electric potential, which has been practically presented via contact angle (precisely advancing contact angle which is particularly employed for LISs/SLIPSs) reducing from 71.5° to 52.9°, evidencing the wide range of wettability gradient in the hydrophilic wetting regime.

The water vapor condensation has been examined on SLI-WGS, as shown in Figure 9(f) [84]. It implies that SLI-WGS is the promising candidate to promote the condensation since the water droplets could easily move and were finally collected at the center driven by the wettability gradient. In addition, the self-cleaning potential of SLI-WGS (Figure 9(g)) is also favorable. The liquid droplet transport in the liquid-liquid system is quite crucial that is also supported by SLI-WGS as presented in Figure 9(h).

SLI-WGS has been attempted on copper sheet, consisting of alternative superhydrophobic strips encased by the liquid-infused hydrophobic patches [85]. The combined method of chemical oxidation, taping, and silicon oil infusion has been adopted. It is important to note here that Li and Guo [85] called that method as chemical etching, which is however a chemical oxidation method because a thin oxidized layer consisting of duplex cupric and cuprous oxides is developed via oxidation reaction among copper, sodium hydroxide, and ammonium persulfate. In applications, SLI-WGSs have been implemented to carry out the droplet and bubble manipulations (directional transport and collection on various tracks). The superhydrophobic strips and LI patches provided the water (average) *θ*_a_ of 156° and 110°, respectively. Since the wetting behavior of bubbles is different compared to that of the droplets, superhydrophobic strips and LI patches provided the bubble (average) *θ*_a_ of 61° and 54°, respectively. In the case of water droplets, the obvious wettability gradient can be seen switching from the superhydrophobic to hydrophobic regime, while the wettability gradient for bubbles changed from high hydrophilic to low hydrophilic regime, which has been considered effective for gas evolution reactions and gas adsorption reactions. For example, the uphill manipulation of a single bubble on straight paths has been successfully reported which was mechanized by the wettability gradient, i.e., a bubble sitting on the superhydrophobic strip is inclined to be on the LI patch and then keeps on moving forward [85].

#### 4.2.2. Slippery Stimuli-Responsive PCM-Infused Wet WGSs

Stimuli-responsive WGSs can be fabricated by infusing the micro/nanostructures with the suitable PCMs that can provide a promising response to thermal, photo, or electric stimuli. However, the literature lacks wet WGSs prepared with PCMs. The governing mechanism of PCM-infused wet WGSs is deemed to be the combination of underlying wettability gradient and the phase gradient of the employed PCMs. Indeed, the switchable wettability across the melting temperatures of PCMs is deemed to be responsible for developing the phase gradients. For example, the full Wenzel state or low adhesion Wenzel state can occur below the melting temperature, while a slippery state can occur above the melting temperature [48]. In another case, temperature-responsive WGS has been presented by infusing PANIPAAM via graft polymerization [86]. The replica of micro/nanoscaffold has been prepared on poly(methyl methacrylate) with the help of a femtosecond laser, which was then transferred to the PDMS through soft-lithography. PANIPAAM responds to temperature and provides three wetting regimes, including the sticky hydrophilic regime below the critical temperature of 30°C, sticky-slippery mixed regime between 30 and 36°C, and slippery superhydrophobic regime above the critical temperature of 36°C. In the mixed regime, the wettability gradient-induced droplet transportation from high water contact angle towards low water contact angle has been successfully depicted. Moreover, the uphill movement of the droplet has also been found. Overall, the novel features of PCMs can provide different ways of tuning the droplet dynamics on the same surface under the influence of active stimuli. Furthermore, the list of PCMs has been provided with the required physical properties in Subsection 5.5.1 that can be equally employed to realize the slippery stimuli-responsive PCM-made WGSs in the future.

Additional wet WGSs are listed in Table 2.

## 5. Bottlenecks and Recommendations

### 5.1. Fabrication Complexities

The fabrication of WGSs is supposed to be the primary key, deciding the potential usage later in device erection either for lab-scale or industrial applications. Commonly, WGSs are created after passing through two steps [61] or multiple steps in a single trial or several different methods are combined and carried out [58, 64]. The fabrication processes, including the plasma treatment, imprinting, vapor deposition, and soft-lithography, become complicated owing to the stringent process constraints or control parameters [12, 41]. For example, a secondary substrate is frequently required to create the footprints on the target substrate, whereas that secondary substrate is often difficult to be designed at the lab scale, thus increasing the fabrication cost when buying it from professional designers. Likewise, high-cost methods such as reactive-ion etching have also been suggested to be replaced by other facile steps [58], as well as the laser cutting and photolithography have been declared complex and time-consuming [85]. Therefore, there is an urgent need to pay special attention on simplifying the fabrication processes to create the robust WGSs by either alternative methods or by modifying the existing methods. Regarding the alternative methods, laser fabrication is strongly suggested which can be selectively controlled, as well as equally feasible for various surfaces including polymeric, ceramics, and glass [12]. In stance of the existing methods, photolithography introduced by Wu et al. [41] is academically useful and technically viable, consisting of an independent photomask (secondary substrate) that can precisely guide the radiations (ultraviolet in their study) towards the target substrate, leading to patterned arrangement in wettability. Their method is capable of creating the desired level of wettability (from superhydrophobic to superhydrophilic regime), which is controllable through irradiated time, i.e., with continuous exposure, the hydrophilic regime keeps on having more wettability that can eventually end at the superhydrophilic wetting regime. Such a method can help avoid the complicated steps encompassing the tight attachment of secondary substrate to the target substrate that may need heat or mechanical stress to leave the footprints, and then, its detachment after generation of footprints is difficult that may need other chemical solutions.

### 5.2. Fear of Liquid-Residue and Pinning

Besides the entire sticky WGSs, an intensive challenge even on slippery WGSs is the possible fear of liquid-residue and pinning in the hydrophilic wetting regime [48]. This is attributable to the intrinsic dynamics of hydrophilicity, giving rise to a filmwise mode of liquid transport, where a thin liquid film tends to remain on the surface even after the flow process is stopped, which can be regarded as the liquid-residue. The liquid-reside can hamper the potential of WGSs in particular applications, for example, condensation heat transfer, fog collection, and water harvesting where the quick liquid transport is necessitated to keep the surface readily fresh for new nucleation sites. Therefore, it is urged to find the robust solutions either by making the hydrophilic regions slippery or by controlling the overall topographic micro/nanostructures such that the wettability gradient can be achieved by keeping the hydrophilic regions as small as possible.

### 5.3. Lifespan of WGSs

The wettability gradient has been found to be greatly influenced by the environment in which WGSs are stored, as well as their storage time is also important. This effect has been investigated by Ta et al. [17], in which the laser treated-brass surface depicted a great change in the apparent contact angles with the passage of time. WGS was eventually converted into superhydrophobic surface over the course of seventeen days. This implies that the shelf-life of WGSs should also be considered as the critical factor and hereby further recommended to experimentally determine the deterioration limits so that WGSs can be effectively utilized within the expiry period.

As the chemical coating on the substrate or the solid structures of the substrate themselves can help create WGSs, the overall lifespan of WGSs is still elusive in terms of the technical information on the structural stability, structural maintenance, operational cyclic reliability, output reproducibility, etc. Chemical coatings are normally more fragile [42]. The economic perspective of WGSs also needs thoughtful actions, which however depends on the total lifespan, encompassing the labor cost, material cost, the periodic cost of structural maintenance, electric cost, etc. It is therefore recommended to deeply investigate WGSs exploring the above features that can grab a great industrial significance in the future.

With a lifespan of WGSs considerably longer than other slippery surfaces (superhydrophobic or SLIPSs), a technological revolution can be brought particularly based on the self-stimulus droplet dynamics necessary for a wide range of applications, including the controllable droplet transport, multiple droplet merging, efficient microfluidics, microreactors, bioassays, microarrays, water harvesting, and condensation heat transfer, as well as in regulating the behavior of gas bubbles important for electrochemical hydrogen and carbon dioxide capturing, transporting, and collection.

### 5.4. Slippery Liquid-Infused WGSs

Until recently, few studies of SLI-WGSs enabled by the electric/gravity stimuli have been presented (WGS obtained by infusing the slippery liquids into their micro/nanoporous scaffold are named as SLI-WGS). SLI-WGSs are almost akin to SLIPSs in providing slippery dynamics. The most interesting trait of SLIPSs is to drive the droplets as efficiently as possible on behalf of the wet oily droplet-surface interaction [89]. However, the surface instability is the biggest challenge encountered by LISs/SLIPSs, which is arisen by the several critical factors, encompassing oil depletion through (1) evaporation [90] which is defined as the self-vaporization of oil at the ambient or high temperature in open space; (2) cloaking [91] in which the oil encapsulates the droplet due to the chemical affinity, and then, it leaves the surface upon droplet shedding; and/or (3) physical shearing [92] that refers to the viscous or frictional impact between the mobile droplet and the infused oil. In a word, the usage of slippery liquids in SLI-WGSs is also deemed to aggravate those challenges. Therefore, the selection and screening of the slippery liquids should be of primary consideration, aiming to avoid or at least alleviate the surface instability bottlenecks. One of the possible ways is to use the right combination of liquid droplet and infused oil, meaning that if the droplet is of water, the infused oil should be completely immiscible with water. Examples of miscible water-oil combinations include the water-silicon oil system, water-GPL oil system (GPL is a label referring to synthetic Krytox oil series classified according to the viscosities), and water-natural oil system (soyabean oil, palm oil, etc.), while the immiscible water-oil combinations include the water-paraffin oil system, water-paraffin waxes system, and water-natural wax system (carnauba wax and beeswax, etc.).

### 5.5. Future Research Directions

#### 5.5.1. Phase Change Materials

Phase change materials (PCMs) are the promising candidates that can exist in various temperature-dependent phases, such as solid, liquid, and mush, as well as in different molecular weights that decide their carbon chain length. Depending on the implemented treatment methods, various dry [93, 94] and wet [95] bioinspired surfaces have been fabricated governed by the temperature, photo, electric, or gravity stimuli, providing the sticky as well as slippery dynamic regimes [96, 97]. Nonetheless, WGSs are deprived of their potential usage at abundant scale. Particularly regarding the bioinspired surface science, the classification of PCMs has been recently presented in waxy and non-waxy branches [98]. The waxy PCMs are categorized into natural (including plant, animal, and mineral waxes) and synthetic (including paraffin oils and paraffin waxes), whereas the non-waxy PCMs are further divided into fatty acids, metals, polymers, salts, and sugar alcohols. The inherent surface properties of PCMs, as tabulated in reference [98], should be kept ahead as the primary design criterion while applying them in surface science.

#### 5.5.2. Evaporative Droplet Dynamics

Studying the droplet dynamics of a droplet resting on the surface under evaporation is of great significance. It not only provides the concrete evidences regarding the sticky and slippery regimes in general, but also the interfacial phenomenon can be inferred by investigating the contact line that is widely known as the contact line dynamics. The contact line dynamics are divided into two categories of constant contact radius (CCR) [99] and constant contact angle (CCA) [100] modes, referring to the sticky and slippery regimes, respectively, as well as the mixed CCR-CCA mode [101]. In addition, the droplet dynamics and the extent of surface adhesion can be quantitatively predicted through the depinning force *F*_d_ (Equation (8) [102]) which is the indication of resistive behavior offered by the surface, as follows:

(8) $$\begin{array}{c} F_{\text{d}}=\gamma_{\text{lv}}\left(\cos\theta_{\text{re}/\text{eva}}-\cos\theta_{\text{ad}/\text{eva}}\right). \end{array}$$

It should be noted that the receding contact angle *θ*_re/eva_ (°) and advancing contact angle *θ*_ad/eva_ (°) belong to the evaporation process, i.e., they should be calculated when the first slip occurs in the base radius of droplets (droplet radius vs. evaporation time graph as can be seen in Reference [94]). Based on such results, the interfacial mechanism of droplet dynamics can be physically understood, particularly at various surface angles, temperatures, and multiple-droplet systems [103, 104]. Nevertheless, evaporative droplet dynamics and contact line dynamics have not been explored on WGSs. It is hereby strongly recommended to carry on such studies that can feasibly reveal the junctional dynamic mechanism of a droplet simultaneously interacting with two wetting regimes, i.e., droplet wetting regime and droplet dynamic regimes can be instantaneously investigated.

#### 5.5.3. Droplet Motion

Although several reports regarding the droplet motion on WGSs have been presented, there is a need to reach the maturity extent. The particular questions and challenges necessitating further efforts include the increased transport velocity, high response rate, and increased transport distance of droplets. Indeed, the droplet velocity is deemed to be affected by the surface hysteresis, wettability gradient range, and wetting regimes. In general, low hysteresis and a large wettability gradient range [42] are indispensable for faster droplet velocity. However, it is highly important to note that the large wettability gradient range may include a sufficient hydrophilic regime, which may negatively affect the droplet velocity due to the dominance of a sticky regime. For example, the reduced droplet velocity on the hydrophilic guided tracks has been reported [70], which is ascribed to the increased droplet surface area. In addition, the presence of nanostructures in the hydrophobic regime of WGSs has suffered from the Wenzel state-pinning effect, which is alarming as it strongly deters the droplet dynamics [26]. Droplet spreading [105] has also been regarded as the hindrance in droplet manipulation on WGSs, because it can drastically influence the droplet dynamics [51]. Therefore, such impacts should also be kept ahead that may be resolved through the efficient fabrication tactics.

## 6. Conclusion

The wettability gradient surfaces (WGSs) have been thoroughly reviewed, particularly shedding a light on the governing principles of wettability gradient and droplet dynamics, their classification, bottlenecks, and future directions. The major inspiration to create artificial WGSs originates from the living surfaces, entirely similar to the fundamental bioinspired surfaces. The quantification criterion of the wettability gradient is supposed to be the spatial derivative of apparent contact angles spanning from the highest (superhydrophobicity) to the lowest (superhydrophilicity) values or in between them, reserving a certain direction. The wettability gradient can be achieved by carefully creating and aligning the topographic micro/nanostructures ensuring the variance of surface energy. However, the efficient droplet dynamics (i.e., droplet mobility) is largely influenced by the topographic micro/nanostructures and surface energy, which is accountable for two dynamic regimes, namely, slippery and sticky. To avoid the sticky dynamic regime, developing the artificial coatings is highly important. WGSs have been categorized in dry and wet surfaces, depending on the apparent surface states. It has been emphasized that the fabrication complexities and the challenges associated with the liquid-residue and droplet pinning especially in the hydrophilic wetting regimes should be addressed in the future. The lifespan of dry WGSs, and in particular, the wet WGSs, needs further efforts. Phase change materials are enlisted and strongly recommended to be employed in the preparation of WGSs so that the phase gradients can be studied. Also, it is recommended to make use of the evaporative droplet dynamics that can effectively imply the transition of a droplet between multiple wetting regimes of WGSs.

Although recent efforts have been seen to promote WGSs, more work on applications is indispensable. For example, the droplet manipulation and droplet size shift seem to be highly influential in water harvesting and condensation heat transfer. The on-demand droplet mobility and droplet control are necessitated in microfluidic systems, as well as in building the novel chemical reactors and electric control systems. Therefore, a large research scope is available in the field of WGSs.

## Figures and Tables

**Table 1:** Pre-investigated dry WGSs.

Ref.	Substrate	Fabrication method	Artificial coating	Stimulus	Gradient regime	Water contact angle	Applications
[16]	Poly(methyl methacrylate) sheet	Laser cutting	PDMS and silica particles	Self	SHPo-HPi	162°-68°	Electrolysis and gas collection
[17]	Brass sheet	Laser texturing	NA	NA	Variable	—	Chemical sensing
[32]	PDMS sheet	Photolithography and plasma etching	NA	Self	HPo-HPo	140°-100°	Underwater air bubble manipulation
[35]	Silicon wafer	Vapor diffusion	Alkyltrichlorosilane	NA	HPo-HPi	100°-52°	Steam condensation and drop distribution shift
[36]	Silicon wafer	Photolithography and dry etching	CYTOP	Self	HPo-HPi	100°-35°	Steam condensation heat transfer
[41]	Glass, cloth, and filter paper	Photolithography and UV treatment	Trimethoxyoctadecylsilane	Self	HPo-HPi	135°-53°	Water droplet manipulation, metering, and merging
[43]	Copper wires	Electrochemical corrosion combined with the gradual solution-rise method	NA	Self	HPo-HPi	NA	Fog collection
[44]	Silicon wafer	Photolithography, reactive, and deep reactive ion etching	Fluoropolymer	Self	SHPo-HPo	150°-118°	Droplet positioning and coalescence-induced motion
[47]	Silicon wafer	Vapor diffusion	Alkyltrichlorosilane	Self	HPo-SHPi	100°-0°	Steam condensation
[50]	Silicon wafer	Vapor diffusion	Dodecyltrichlorosilane and decyltrichlorosilane	Self	HPi-HPi	70°-10°	Tetraethylene glycol droplet manipulation
[51]	Graphite plate	Electrochemical oxidation	PCM (paraffin wax)	Self	HPo-HPi	104°-31°	Water droplet manipulation
[52]	Glass plate	Vacuum ultraviolet-assisted photodegradation	Octadecyltrimethoxysilane	Self	HPo-HPi	NA	Water droplet manipulation
[53]	Acrylic polymer	Ultraviolet-ozone treatment	Fluorosilane	Self	HPo-HPi	120°-60°	Water collection
[54]	Graphite plate	Electrochemical oxidation combined with gradual solution removal method	NA	Gravity	HPo-HPi	123°-66°	Underwater oil droplet manipulation
[57]	Copper plate	Laser cutting and vapor deposition	Perfluorodecanethiol	NA	SHPo-HPo	172°-131°	Steam condensation heat transfer
[58]	Cyclic olefin copolymer and thermoplastic polymer	Interference lithography, UV, and thermal-nanoimprint lithography	Perfluorodecyltrichlorosilan	NA	HPo-HPo	132°-108°	NA
[62]	Silicon wafer	Photolithography, deep reactive ion etching, and oxygen-based plasma treatment	Fluoropolymer	Self	SHPi-HPo	0°-105°	Droplet splitting
[63]	Silicon	Optical lithography	Perfluorodecyltrichlorosilane	NA	HPo-HPi	128°-70°	Glycerol/water droplet transport
[64]	Silicon wafer	Photolithography and deep reactive ion etching	Perfluorooctyl silane	NA	HPo-HPi	107°-67°	Water vapor condensation
[65]	Silicon wafer	Photolithography, plasma etching, and gradual solution rise	Decyltrichlorosilane	Self	SHPo-HPi	151°-39°	Water droplet manipulation
[66]	Copper mesh and copper sheet	Femtosecond laser scanning and vapor deposition	Polytetrafluoroethylene (sheet)	Gravity	SHPo-HPi	159°-76°	Fog harvesting
[67]	PDMS	Laser irradiation	NA	Vibration and gravity	HPo-HPi (periodic)	125°-NA	Droplet manipulation, biochemical detection
[68]	Cu mesh combined with PDMS-graphene	Laser etching and ultrasonic vibration	NA	NA	SHPo-HPo	153°-117°	Fog collection
[69]	Janus polyester/nitrocellulose textile	CO_2_-based laser drilling	NA	NA	HPi-SHPo	84°-0°	Sweat droplet transportation and thermal management of the human body
[70]	Silicon nanowires	UV-enhanced photodecomposition	Dodecyltrichlorosilane	Gravity	SHPo-HPi	150°-NA	Guiding droplet tracks
[71]	Glass slide	Laser ablation	Polydimethylsiloxane and silica	Gravity	SHPo-HPi	153°-34°	Underwater gas bubble manipulation
[72]	Aluminum plate	Gradual substrate move and vapor deposition	Perfluorodecyltrichlorosilane	Self	HPo-HPi	—	Steam condensation heat transfer
[73]	Silicon wafer	Gradual substrate move (layer by layer deposition)	Polyelectrolytes	NA	SHPo-SHPi	164°-5°	Rewritable and switchable wetting
[74]	Aluminum plates	Physical vapor deposition	Heptadecafluoro-1-decanethiol	Self and magnetic	HPo-HPi	90°-15°	Ferrofluid droplet manipulation
[75]	Polyethylene	Oxidation (electrode corona treatment)	NA	NA	HPo-HPi	95°-45°	Cell growth and protein adsorption
[76]	Glass	Vapor deposition(moving substrate)	Octadiene-allyalamine	NA	HPo-HPi	90°-70°	Protein adsorption and cell culturing
[77]	Polyethylene sheets	Corona oxidation (moving substrate)	NA	NA	HPo-HPi	100°-40°	Platelet adhesion and protein adsorption
[78]	Polyethylene sheets	Corona oxidation (moving substrate)	Monomer solution	NA	HPi-HPi	90°-40°	Platelet adhesion
[79]	Coverslips	Plasma polymerization	Octadiene-acrylic acid	NA	HPo-HPi	96°-48°	Cell culturing and adhesion
[80]	Silicon wafer	Photolithography	NA	Self	NA	NA	Water droplet manipulation
[81]	Aluminum alloy	Electrochemical etching and electrolyte jet machining	PCM (stearic acid)	Gravity	NA	NA	Droplet adhesion and sliding
[82]	Nickel-titanium sheet	Laser etching	PCM (stearic acid)	Self	SHPo-HPo	155°-94°	Antithrombosis, antiadhesion of protein and cells
[83]	Aluminum sheet	Laser scanning (patterned arrangement)	PCM (stearic acid)	Self	SHPo-Hpo	161°-98°	Underwater air bubble manipulation

Abbreviations: SHPo: superhydrophobic; HPo: hydrophobic; SHPi: superhydrophilic; HPi: hydrophilic; NA: not available.

**Table 2:** Pre-investigated wet WGSs.

Ref.	Substrate	Fabrication method	Artificial coating	Stimulus	Gradient regime	Water contact angle	Applications
[13]	Glass wafer	Photolithography, reactive ion etching, and oil infusion	Salinized nanoparticles and silicon oil	Self	SHPo-HPo	165°-108°	Water droplet manipulation (uphill and straight mobility)
[84]	Glass	Printing and oil infusion	Krytox GPL-103 oil	Electric potential	HPi-HPi	71.5°-52.9°	Underoil water droplet manipulation, water condensation, and dust-cleaning
[87]	Glass wafer	Photolithography and oil infusion	Ionic liquid and Krytox GPL oil	Self	NA	NA	Water and ethylene glycol droplet manipulation
[88]	Silicon wafer	Vapor diffusion	Chlorine-terminated polydimethylsiloxane	Self	HPi-HPi	26.9°-21.5° (FC40)	Manipulation of low surface tension fluids and their condensation

Abbreviations: SHPo: superhydrophobic; HPo: hydrophobic; SHPi: superhydrophilic; HPi: hydrophilic; NA: not available.

**Table 3:** Potential PCMs and their melting temperatures [24].

PCMs	*T*_m_ (°C)	PCMs	*T*_m_ (°C)
Waxy paraffin oils			
RT-(-9) HC	-9	Tetradecane	5-6
RT-(-4)	-4	RT-8	8
RT-0	0	Pentadecane	10
RT-2 HC	2	RT-12	12
RT-3 HC_1	3	RT-15	15
RT-4	4	Hexadecane	18-19
Paraffin waxes			
Heptadecane	22	Tricosane	48-50
Octadecane	28	Tetracosane	50-52
Nonadecane	32-33	Pentacosane	52-54
Eicosane	36-37	Hexacosane	56-68
Heneicosane	39-41	Heptacosane	58-60
Docosane	42-44	Octacosane	60-62
Waxy natural PCMs			
Peanut oil	2-3	Carnuaba wax	80
Coconut oil	25	Palm oil	—
Cocoa oil	35	Tea wax	—
Beeswax	61		
Nonwaxy fatty acids			
Butyric acid	-5.6	Pentadecanoic acid	52-53
Caproic acid	-3	Palmitic acid	61-64
Caprylic acid	16-17	Margaric acid	60
Capric acid	30-32	Stearic acid	65-70
Lauric acid	41-44	Nonadecylic acid	67
Tridecylic acid	41.4	Heneicosylic acid	73-74
Myristic acid	49-58	Tricosylic acid	79
Nonwaxy polyalcohols			
Threitol	88.6	Erythritol	117-120
Xylitol	92-96	D-Mannitol	166-167
Adonitol	96-104	Dulcitol	187-189
Sorbitol	93-99	Inositol	224
Arabinitol	106.2		
Nonwaxy polymers			
PEG400	3.2	PEG3400	56.5
PEG600	22.2	PEG4000	59.7
PEG1000	32	PEG6000	64.8
PEG1500	46.5	PEG10000	66
PEG2000	51	PEG20000	68.7
PANIPAAM	32-36		
Nonwaxy salts and salt hydrates			
AlCl_3_	192	MgCl_2_	714
LiNO_3_	250	Na_2_P_2_O_7_·10H_2_O	70
NaNO_3_	307	Ba(OH)_2_·8H_2_O	78
KNO_3_	333	(NH_4_)Al(SO_4_)_2_·12H_2_O	95
KOH	380	MgCl_2_·6H_2_O	117
KclO_4_	527	Mg(NO_3_)_2_·6H_2_O	89.3
LiH	699		
Nonwaxy metals			
Mercury	-38.8	Lithium	186
Cesium	28.6	Sn_91_Zn_9_	199
Gallium	29.8	Tin	232
Bi_58_Sn_42_	138	Bismuth	271.4
